# Prioritizing Trust in Podiatrists’ Preference for AI in Supportive Roles Over Diagnostic Roles in Health Care: Qualitative Interview and Focus Group Study

**DOI:** 10.2196/59010

**Published:** 2025-02-21

**Authors:** Mohammed A Tahtali, Chris C P Snijders, Corné W G M Dirne, Pascale M Le Blanc

**Affiliations:** 1 Department of Industrial Engineering & Management Fontys University of Applied Sciences Eindhoven The Netherlands; 2 Department of Industrial Engineering & Innovation Sciences Human Technology Interaction group Eindhoven University of Technology Eindhoven The Netherlands; 3 Department of Industrial Engineering & Innovation Sciences Human Performance Management group Eindhoven University of Technology Eindhoven The Netherlands

**Keywords:** AI’s role in health care, decision-making, diabetes and podiatrists, trust, AI, artificial intelligence, qualitative, foot, podiatry, professional, experience, attitude, opinion, perception, acceptance, adoption, thematic, focus group

## Abstract

**Background:**

As artificial intelligence (AI) evolves, its roles have expanded from helping out with routine tasks to making complex decisions, once the exclusive domain of human experts. This shift is pronounced in health care, where AI aids in tasks ranging from image recognition in radiology to personalized treatment plans, demonstrating the potential to, at times, surpass human accuracy and efficiency. Despite AI’s accuracy in some critical tasks, the adoption of AI in health care is a challenge, in part because of skepticism about being able to rely on AI decisions.

**Objective:**

This study aimed to identify and delve into more effective and acceptable ways of integrating AI into a broader spectrum of health care tasks.

**Methods:**

We included 2 qualitative phases to explore podiatrists’ views on AI in health care. Initially, we interviewed 9 podiatrists (7 women and 2 men) with a mean age of 41 (SD 12) years and aimed to capture their sentiments regarding the use and role of AI in their work. Subsequently, a focus group with 5 podiatrists (4 women and 1 man) with a mean age of 54 (SD 10) years delved into AI’s supportive and diagnostic roles on the basis of the interviews. All interviews were recorded, transcribed verbatim, and analyzed using Atlas.ti and QDA-Miner, using both thematic analysis for broad patterns and framework analysis for structured insights per established guidelines.

**Results:**

Our research unveiled 9 themes and 3 subthemes, clarifying podiatrists’ nuanced views on AI in health care. Key overlapping insights in the 2 phases included a preference for using AI in supportive roles, such as triage, because of its efficiency and process optimization capabilities. There is a discernible hesitancy toward leveraging AI for diagnostic purposes, driven by concerns regarding its accuracy and the essential nature of human expertise. The need for transparency and explainability in AI systems emerged as a critical factor for fostering trust in both phases.

**Conclusions:**

The findings highlight a complex view from podiatrists on AI, showing openness to its application in supportive roles while exercising caution with diagnostic use. This result is consistent with a careful introduction of AI into health care in roles, such as triage, in which there is initial trust, as opposed to roles that ask the AI for a complete diagnosis. Such strategic adoption can mitigate initial resistance, gradually building the confidence to explore AI’s capabilities in more nuanced tasks, including diagnostics, where skepticism is currently more pronounced. Adopting AI stepwise could thus enhance trust and acceptance across a broader range of health care tasks, aligning technology integration with professional comfort and patient care standards.

## Introduction

### Background

As artificial intelligence (AI) continues to advance, its potential to revolutionize health care is unquestionable. AI systems are increasingly capable of performing tasks that range from routine data analysis to complex decision-making processes that were once the sole domain of human experts. These technologies offer the promise of more accurate predictions [1], more precise patient condition explanations [2], autonomous diagnoses [3], and personalized treatment plans [4]. Despite these capabilities, there remains an evident hesitancy among health care professionals and patients alike to embrace AI-driven recommendations fully. This skepticism is partly rooted in worries about AI’s capacity to grasp patients’ nuanced, individual scenarios [5], to integrate the crucial human elements of empathy and understanding [6,7], and to deal with complex ethical considerations in health care [8].

The literature on trust in AI highlights concerted efforts to foster trust by exploring strategies to address trust issues between AI systems and users. For example, some studies suggest that redefining AI as a supportive adjunct to professionals rather than a standalone solution could promote acceptance [9,10]. In addition, showcasing instances where AI surpasses human experts in specific tasks may enhance humans’ trust in AI [5,9,11]. Emphasizing transparency and providing understandable explanations of AI processes are crucial, with evidence showing users how AI works can greatly diminish skepticism [12-15]. For example, applying explainable AI (XAI) techniques to explain the basis of AI-generated predictions can, in certain contexts, enhance human trust and understanding [12]. Although XAI can potentially be valuable in increasing trust and understanding, the specific implementation of XAI (eg, the complexity and relevance of the explanation) is crucial in achieving those goals [16]. However, despite these positive developments, fully integrating AI into practice remains challenging, and professionals and laypersons often still prefer human judgment and intuition over AI recommendations [6,7,10,17].

AI’s potential applications within health care are vast and multifaceted. Its well-established ability to detect diseases such as cancer and predict various health conditions highlights its substantial role in diagnostics. Not only does AI excel in recognizing patterns within medical images for early disease detection, offering a level of precision that can sometimes surpass traditional methods [3,18,19], but also its influence extends beyond diagnostics. AI assumes a crucial supportive role in health care operations, such as triaging in emergency departments where algorithms prioritize care based on urgency and outcomes, thereby optimizing resource allocation and ensuring timely patient attention [20]. Furthermore, AI’s capacity for enhancing operational efficiency is evident across a spectrum of health care activities. It predicts bed availability and automates the conversion of patient consultations into concise summaries, thereby freeing health care professionals from administrative work. This enables more patient-centered care and marks AI as a transformative tool in health care [21].

This study seeks to delve deeper into health care providers’ perspectives on AI’s current and potential role and value in their field. While previous studies often focus on assessing trust in already developed AI systems, this study takes a different approach by involving health care professionals from the outset in identifying potential applications of AI. Early engagement allows us to determine where AI is anticipated to be most valuable, fostering a more collaborative and user-centered integration that aligns with professionals’ needs and expectations. By engaging directly with health care professionals who are the prospective users of these AI applications, the study seeks to pinpoint areas where AI integration would be most advantageous and eagerly anticipated by practitioners. Through this inquiry, we aspire to pave the way for more effective and user-endorsed integration of AI in health care, aligning technological advancements with health care professionals’ real-world needs and preferences.

### Objective

Our study explores health care providers’ perceptions of AI’s potential benefits in patient care and operational efficiency, with examples including risk assessments and diagnostics. The effectiveness of AI technologies depends on their advanced capabilities and, more crucially, on their acceptance by health care professionals. Without their acceptance, achieving a truly successful implementation becomes only partial, leading to the largely unrealized potential of AI. Thus, we aim to understand these professionals’ perspectives on AI adoption, emphasizing the development of trust and integration strategies and identifying approaches that position AI as a valuable tool complementing human expertise, not replacing it [10]. Consequently, the central research question we propose is as follows: How can AI be effectively integrated into practice in a way that ensures health care professionals’ acceptance and trust in its advice?

We adopt a qualitative approach to address our research question, focusing on the FootCheck (RondOm Podiatrists) app (in Dutch: Voetencheck app). This app, specifically designed for the daily monitoring of patients with diabetes in the Netherlands, sends daily push notifications to guide these patients through a foot screening process, featuring instructional videos and questions. This enables patients to submit photos to podiatrists to evaluate potential issues such as wounds or ulcers. During its successful pilot phase, which occurred amid the COVID-19 pandemic, the app was used by over 1700 individuals, leading to more than 43,000 screenings [22]. Recognizing the growing interest of stakeholders in leveraging AI to enhance efficiency and health care quality, we believe the FootCheck app provides an ideal basis for exploring AI’s potential. It is important to note that the app still lacks AI integration, highlighting the potential benefits of AI for future enhancements. Our study includes interviews with diverse podiatrists, encompassing current and potential future app users. These interviews aim to understand their experiences and perspectives regarding using the app as it is and the prospects for integrating AI into the screening process of future versions of the app.

## Methods

### Design and Setting

**Figure 1:**
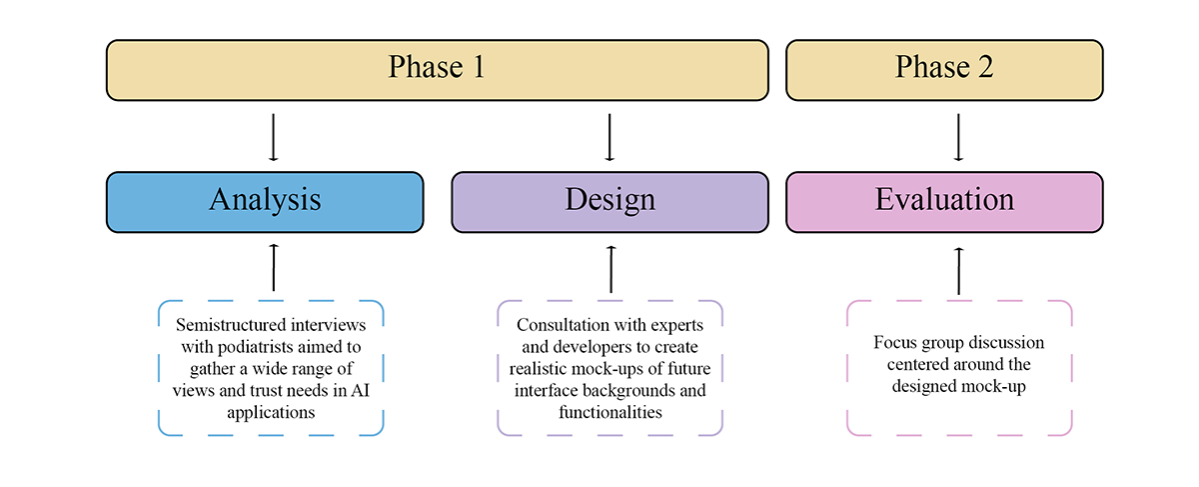
A visual presentation of the 2 phases of the study. AI: artificial intelligence.

We conducted a qualitative study structured in 2 phases to investigate trust and acceptance in AI. In phase 1, we sought to capture diverse perspectives and needs from podiatrists regarding their trust in and acceptance of AI technologies through semistructured interviews. The insights gathered from these interviews served as input to the mock-up design process. Phase 2 aimed to further explore specific AI applications through a focus group session. Figure 1 provides a visual representation of these phases.

### The FootCheck App and Decision-Making Process

**Figure 2:**
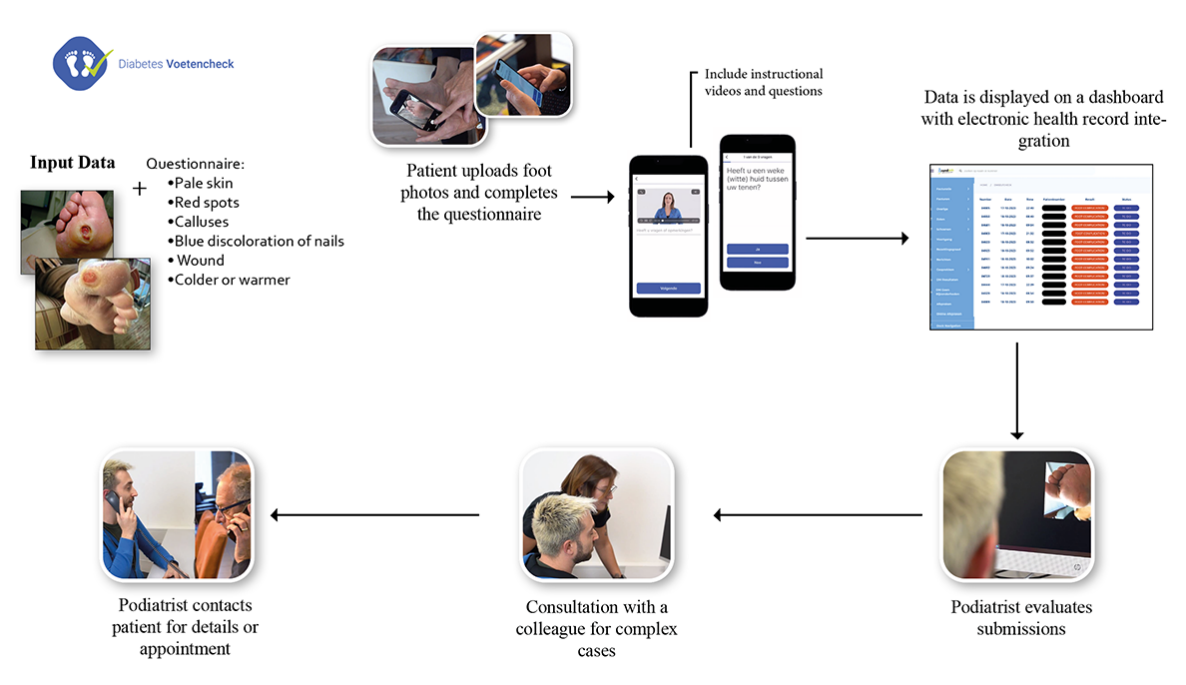
Workflow from patient submission to professional evaluation.

The practical application of AI in health care settings we studied was the FootCheck app, developed for RondOm Podiatrists. As one of the largest podiatry chains in the Netherlands, RondOm Podiatrists introduced this app during the COVID-19 pandemic to ensure continuous care for their patients. The app has since been used to facilitate daily monitoring of foot health for patients with diabetes, allowing patients to submit photographs for screening. The workflow from patient submission to professional evaluation is illustrated in Figure 2.

RondOm Podiatrists are keenly interested in the potential of AI to enhance their app and streamline decision-making processes. The system does not use AI currently, but there are numerous potential areas where AI could provide substantial benefits. For example, AI could be instrumental in assessing the suitability of submitted photos for evaluation. This capability would significantly reduce the workload on professionals by automatically filtering out low-quality or irrelevant images, ensuring that only those suitable for review are presented. This efficiency gain is crucial as it allows podiatrists to focus their expertise where it is most needed rather than on preliminary sorting tasks.

Moreover, AI could assist in prioritizing cases on the basis of urgency. Currently, podiatrists must manually open and evaluate all submitted photos, which is time-consuming and potentially less effective in quickly identifying urgent cases. By implementing AI to perform a preselection of instances based on predetermined criteria of urgency, the system could ensure that patients requiring immediate attention are identified and prioritized, thereby optimizing patient care and resource allocation.

Another critical area in which AI could have a significant role is aiding in the diagnosis of foot conditions. The increasing number of patients with diabetes and a relative shortage of podiatrists present a growing challenge. AI can alleviate some of this burden by providing preliminary assessments based on the analysis of photos. While the professional community may have reservations about relying entirely on AI for diagnoses, its role in offering support and preliminary assessments could be invaluable. This assistance could help bridge the gap in service provision, ensuring that more patients receive timely and effective care.

### Phase 1: Participants and Recruitment Process

In the first phase of our study, we engaged in interviews with 9 podiatrists from 4 distinct practices, aiming to gather diverse insights into the use and perception of the health care app in question. The actual use of the current app varied considerably among participants, encompassing some who used the app daily and others who, while not yet current users, were familiar with its functionalities. Furthermore, participants’ professional expertise spanned different aspects of podiatric care, including both foot care for patients with diabetes and general foot health services.

The recruitment of participants was facilitated through a collaborative effort with the Fontys University of Applied Sciences, one of the leading institutions for podiatry education in the Netherlands. This collaboration was essential for 2 reasons. First, Fontys University maintains strong ties with its alumni, many of whom stay actively engaged with the university through professional networks, alumni events, and workshops. Second, Fontys University’s Health Innovations & Technology research group maintains regular collaborations with health care institutions, ensuring strong connections within the field. Through these partnerships, we recruited a diverse group of motivated podiatrists who voluntarily participated in the study. By using Fontys University’s network and its respected position within the field, we ensured the participation of podiatrists with varying levels of experience and expertise across different areas of podiatric care, contributing to a representative sample for our study. To accommodate our participants’ varying schedules and preferences, the interviews were conducted using a hybrid approach; some were held in person, taking advantage of the natural setting of the participants’ practice environments, while others were carried out through the Teams (Microsoft Corp) platform.

### Data Collection

We started an extensive literature review on trust in AI, both generally and within the health care context, from which we identified key topics. These topics were categorized into 4 groups as follows: knowledge of AI, trust in humans and AI advice, AI’s added value to daily tasks, and factors affecting trust in AI. On the basis of these categories and codes, we formulated initial interview questions. Next, we sought feedback on these initial questions from experts in human and machine interaction. We incorporated their feedback and refined the questions to ensure their relevance and comprehensiveness. To validate the clarity and comprehensiveness of these questions, we conducted a preliminary interview with an experienced podiatrist. This pilot interview served to ensure the questions were understandable and relevant for the prospective interviewees, and it also provided an opportunity to identify any missing questions from the practical perspective of podiatry. The resulting interview guide is detailed in Multimedia Appendix 1.

Participants were briefed on the study’s objectives—namely, to explore their perceptions of AI, identify factors influencing trust in AI among podiatrists, examine the impact of trust on AI adoption, and assess their needs for trusting AI in patient care. They were requested to share demographic information via a link before the interview sessions. The specific interview questions were withheld in advance to preserve the authenticity of responses. The interviews were conducted from July to September 2023.

Consent for recording was obtained from all podiatrists, with the sessions captured either on a voice recorder or through the Teams platform, depending on the interview format. These recordings were transcribed verbatim, using Amberscript [23] for efficiency and accuracy. For interviews conducted on the Teams platform, we leveraged the platform’s transcription functionality. Each interview spanned approximately 30 to 45 minutes, with podiatrists having provided explicit consent, either written or digital, for participating and recording their responses. They were also assured of their right to withdraw from the study at any time.

### Data Analysis of the Semistructured Interviews

We used a dual-coding strategy, where 2 researchers independently analyzed the interview transcripts to ensure a robust examination of the data. Researcher 1 (MAT), a PhD candidate with a solid background in qualitative research methodologies, and researcher 2 (Ruben Gloudemans) used distinct software tools for coding—Atlas.ti (version 11; Atlas.ti Scientific Software Development GmbH) and QDA-Miner (version 6.0; Provalis Research). This divergence in tool use stemmed from their prior experiences and the software licenses available.

Following the thematic analysis by Braun and Clarke [24], we initiated our analysis by thoroughly reading the transcripts to gain an initial understanding and verify their accuracy. First, we labeled the transcripts without predefined categories, using an inductive approach leading to an extensive list of initial codes directly derived from the data. For example, codes such as human interaction, urgency ranking, efficiency, risk detection, and false advice were formed. We then examined these initial codes to determine if they overlapped with the topics we had previously identified from the literature and combined them if needed. For example, urgency ranking and risk detection were put together since both are related to the topic added value of AI. In this step, we started a deductive approach, where we applied existing theoretical frameworks to refine our codes into coherent themes. We repeated this analytical process several times, refining our approach each time to ensure the emergence of concrete themes. This process yielded a preliminary set of themes.

Subsequently, our individually identified themes were compared in a meeting, discovering significant overlap. For some themes where our interpretations initially diverged, we reached consensus through discussion, using inductive and deductive reasoning. These discussions revealed that, despite using different terminologies, we were referring to the same thematic content. Our thematic analysis was refined further through this iterative process of inductive and deductive coding.

### Mock-Up Design

Guided by our research question on how AI can effectively integrate into the FootCheck app’s existing screening process with user acceptance, we sought the podiatrists’ insights on how they would like to receive advice and in which forms to ensure their confidence in it. Various suggestions emerged, such as explaining the reasoning behind the advice, photos pinpointing potential complications on the feet, and color-coding systems for triage. However, the first phase did not conclusively determine which forms of applications and the level of transparency would foster greater trust.

To further explore these possibilities, we organized a focus group featuring mock-ups. Presenting a realistic representation was crucial, particularly considering that currently, the app and its screening process do not use AI.

1. The first mock-up was designed to assess the effectiveness of AI in a supportive role, specifically in scheduling and prioritizing patient consultations based on an array of time indicators.
2. The second mock-up aimed to explore the potential of AI in diagnosing conditions by analyzing photographs submitted by users and identifying common foot complications such as ulcers or calluses.

We aimed to delve deeper into assessing these AI roles by examining their functionality and acceptability among podiatrists. We crafted 3 distinct design variations for each mock-up to achieve this, guided by needs and concerns expressed in the first interviews with the podiatrists. Our primary reason for this approach was to facilitate a broad discussion by presenting transparency levels varying from low to medium to high. This method allowed us to not only compare the discussions across different AI applications but also delve into the discussions within each level of transparency. Thereby, we aimed to explore the range in podiatrists’ perceptions and acceptance extensively.

**Figure 3:**
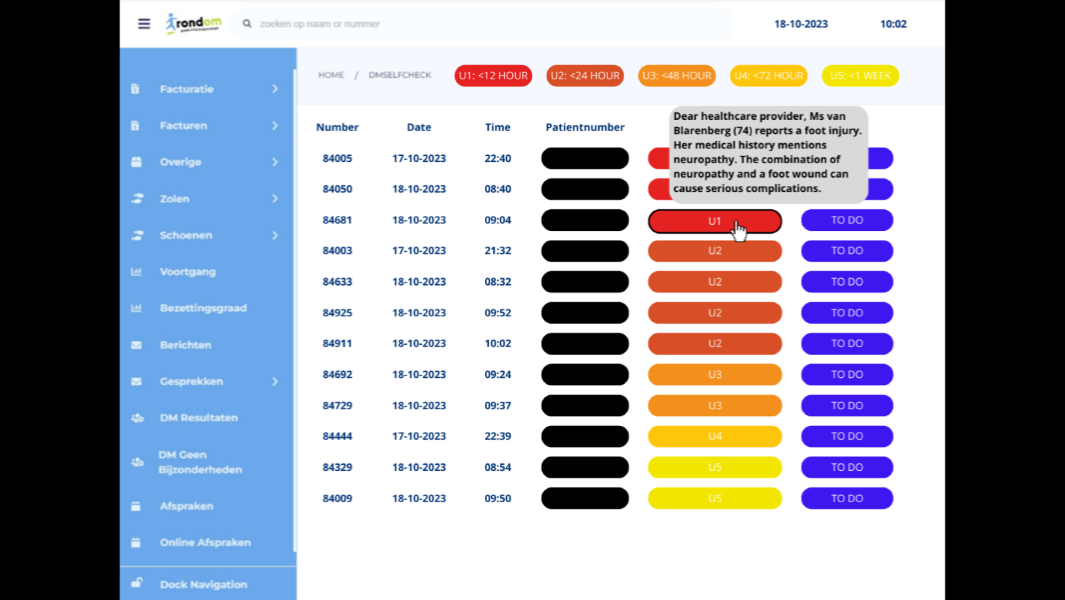
Mock-up 1: high-transparency variant.

**Figure 4:**
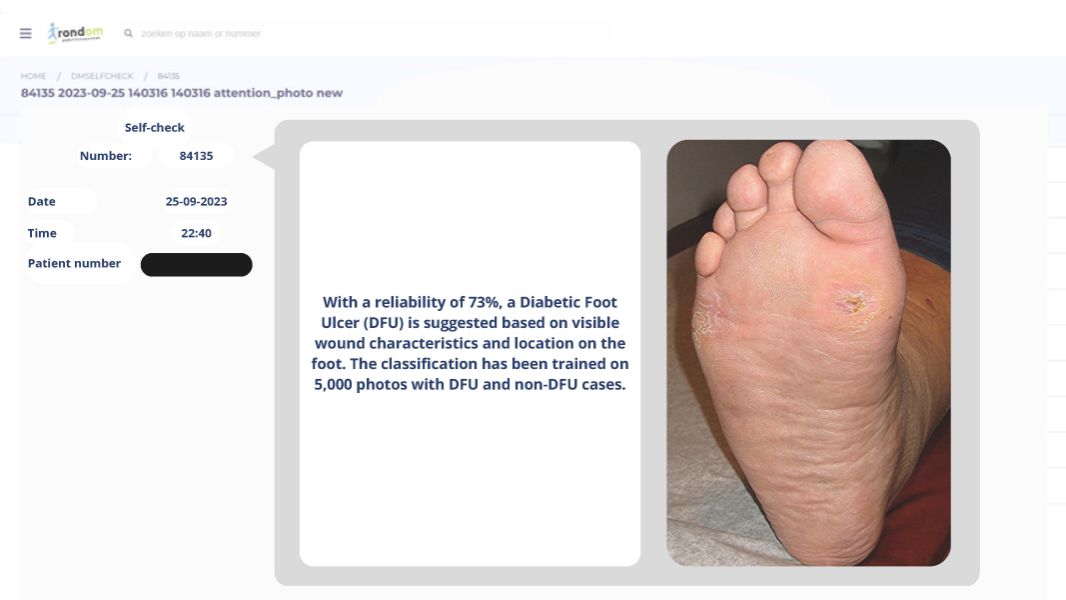
Mock-up 2: medium-transparency variant.

In designing these mock-ups, we used the current interface environment to ensure familiarity for users. In addition, we consulted with experts during the design process to ensure the mock-ups were practical and reflective of current best practices in the field. For an illustration of mock-up 1 with a high transparency level, see Figure 3; for the diagnostic mock-up set in a medium transparency, refer to Figure 4. For a detailed overview of all variants, refer to Multimedia Appendix 2.

### Phase 2: Participants and Recruitment Process

Phase 2 was aimed at enriching our understanding and validating the outcomes of the first phase. Initially, we used the same approaches that had effectively recruited participants for phase 1, including relying on Fontys University’s network and existing partnerships within the podiatry field. We also reached out to contacts from our previous interviews. However, these efforts alone were insufficient to meet our needs for new participants for phase 2. To broaden our reach, we engaged with potential candidates on social media platforms such as LinkedIn (LinkedIn Corp) and Facebook (Meta Platforms, Inc) and attended podiatrist conferences to connect with new professionals. Through these combined efforts, we recruited 4 new podiatrists who had not been involved in the first phase and reinvited one participant from phase 1. The focus group session was organized at the Fontys University of Applied Sciences’ inspiration space in December 2023.

### Procedure for Focus Group and Data Collection

To evaluate our mock-ups, we created an interview guide on essential topics derived from the findings of phase 1: trust in AI’s role, transparency preferences, and AI’s effect on decision-making, outlined in Multimedia Appendix 3. During the focus group session, participants were thoroughly briefed on the structure of the session that was divided into two 45-minute segments with a break in between. The first session explored mock-up 1, while the second delved into mock-up 2.

The roles of MAT and Ruben Gloudemans within the session were clearly defined for participants; MAT served as the observer, summarizing discussions and posing questions, whereas Ruben Gloudemans led the session and presented the materials. Participants provided written consent by signing a consent form.

After a brief introduction, participants were provided two A3-sized sheets, each showcasing 3 mock-up variants highlighting different transparency levels. Before initiating the group discussions, participants were asked to individually rank these variants from 1 to 3, with 1 being their top preference, and to write down comments next to each variant explaining their choices. This step was designed to minimize mutual influence and ensure that each participant’s initial reactions and thoughts were captured independently. Following this individual reflection, detailed group discussions were held, focusing on participants’ rationale regarding their preferences and trust in the AI advice shown in each variant.

### Data Analysis Focus Group

All participants attended in person for the data collection of phase 2. Initially planned as two 45-minute segments with a 15-minute break in between, the session ultimately lasted 70 minutes in a single stretch. This was shorter than anticipated, primarily because the first segment was concluded earlier than expected, and participants opted to proceed without taking the scheduled break.

We adopted a more structured coding method as we progressed to the second phase, building on the codes and themes identified in the first phase. This shift was enabled by implementing a thematic framework approach, a systematic strategy that helped us to systematically organize and interpret the data [25].

After verifying transcription accuracy and familiarizing ourselves with the transcript, we developed an analytical framework. This framework included predefined themes from phase 1: AI’s roles, transparency levels, data reliability, trust development, and the significance of human interaction. This step laid the groundwork for indexing, where we systematically tagged the data with our framework’s themes. Next, through charting, we distilled participants’ responses into structured summaries, aligning them with the thematic framework. In the indexing and charting phase, we organized the data into themes linked to our research goals, creating visual charts. This approach helped us spot patterns and differences, uncovering insights that enhanced our understanding.

### Ethical Considerations

No medical devices were used in the studies, and no physical interventions were performed on participants. The image used in mock-up 2 originated from publicly available internet sources and was appropriately licensed. Consequently, under regulations stipulated by the Dutch Medical Research Involving Human Subjects Act (WMO), the necessity for ethics approval from a national ethics committee was circumvented. However, ethical clearance was proactively obtained from the internal ethics committee of Fontys University of Applied Sciences, documented under project numbers 050324 and 080324. There were no conflicts of interest between the participants and the FootCheck app. None of the participants had any financial, developmental, or other professional ties to the app.

## Results

### Phase 1: Semistructured Interviews

#### Participants

Participant demographics for phase 1 are listed in Table 1. In total, 9 individuals, all with backgrounds in podiatry, participated in the first phase of our study. A distinguishing characteristic of this cohort is their active engagement in clinical practice, that is, their extensive practical experience.

**Table 1:** Participants’ characteristics from semistructured interviews in phase 1 (n=9).

Characteristics			Participants, n (%)
**Sex**			
	Female	7 (78)	
	Male	2 (22)	
**Age group (y)**			
	20-30	1 (11)	
	31-40	2 (22)	
	41-50	3 (33)	
	51-60	2 (22)	
	61-70	1 (11)	
**Education level**			
	Bachelor’s	4 (44)	
	Master’s	4 (44)	
	PhD	1 (11)	
**Specialized education: foot care for patients with diabetes**			
	Yes	4 (44)	
	No	5 (56)	
**Experience (mo)**			
	0-6	1 (11)	
	6-12	2 (22)	
	12-24	4 (44)	
	24-36	2 (22)	
**Experience with the app**			
	Yes	2 (22)	
	No	7 (78)	

#### Themes From the Interviews

**Figure 5:**
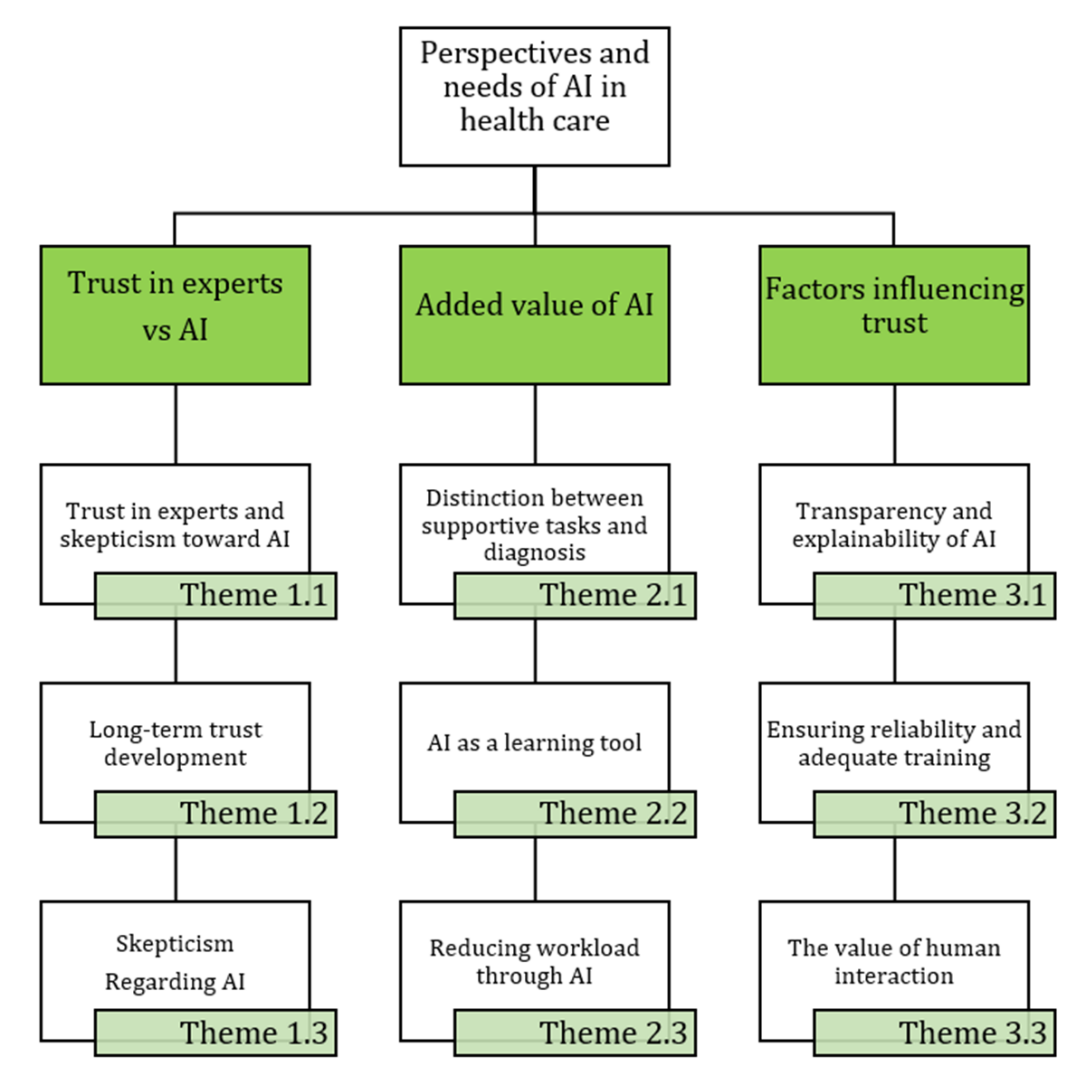
Generated themes and subthemes from phase 1: perspectives of experienced podiatrists on the FootCheck app and decision-making process. AI: artificial intelligence.

The interviews with podiatrists revealed 3 critical themes regarding podiatrists’ perspectives and requirements for AI integration in the FootCheck app and related decision-making process: trust in human experts versus in AI, the added value of AI, and the factors influencing trust in AI. Each theme is further divided into 3 subthemes. A detailed overview of these key themes can be found in Figure 5.

#### Theme 1: Trust in Experts Versus AI

In the initial phase of the interview, we began by asking participants about their understanding of AI and their trust in it compared with their colleagues (ie, experts). We aimed to uncover how AI impacts their trust levels and the underlying reasons for these views.

### Theme 1.1: Trust in Experts and Skepticism Toward AI

Podiatrists expressed trust in the advice from expert colleagues, on the basis of their extensive knowledge and experience. They recognized that making mistakes is part of being human and considered it normal for colleagues to err every now and then. Conversely, when receiving advice from AI, direct trust is not immediately established for most podiatrists. They maintained a critical stance, emphasizing the need to verify the accuracy of AI-generated advice:

I trust a colleague, sometimes blindly, because they have much experience.

Participant 7, female, age group 61-70 years

I see the added value of AI advice. But I still want to check it or have something alongside it.

Participant 2, female, age group 51-60 years

I do have trust in advice from colleagues. I’ve considered practical situations, and nine times out of ten, I agree with them just fine. And there are always nuanced differences because you see things slightly differently with your experience, perspective, and view. But let me put it this way: I firmly trust it.

Participant 8, male, age group 41-50 years

### Theme 1.2: Long-Term Trust Development

Participants indicated that AI may initially struggle to provide accurate advice due to the limited data available and the learning phase required for the model. They also mentioned their initial lack of experience with using AI, which leads to uncertainty about its performance. However, participants felt their trust can increase as they gain more experience with the AI model:

In the beginning, I was sceptical, but as it sees and evaluates more photos, my trust grows.

Participant 9, female, age group 20-30 years

Yes, AI needs to prove itself first, and then it can add value.

Participant 7, female, age group 61-70 years

Indeed, my trust in AI would significantly increase if, over time, the technology evolves to a stage where the AI system is fully operational and applicable.

Participant 6, female, age group 41-50 years

### Theme 1.3: Skepticism Regarding AI

Participants expressed significant concerns about the potential errors an AI system might make and the severe consequences that could arise from such mistakes. In addition, they highlighted a notable limitation of AI: its inability to conduct physical examinations on patients. Such examinations are often essential for a thorough understanding of a patient’s foot health, indicating a gap in AI’s capabilities in health care settings:

Yes, if a patient says: “I have pain in my foot, for example, in the ankles.” Well, AI can’t see that or physically feel or move it.

Participant 9, female, age group 20-30 years

We’re not talking about a patient getting advice to buy Shoe A, and then they say it’s too big and buy another shoe.... We’re talking about serious outcomes, like the loss of a foot, a leg, or even a life, and that’s a significant responsibility.

Participant 4, female, age group 31-40 years

The consequences are quite significant if you miss something there.

Participant 7, female, age group 61-70 years

#### Theme 2: Added Value of AI

### Overview

In exploring AI’s potential impact on podiatry, we engaged participants in discussions about integrating AI into their everyday practice. This dialogue revealed 3 insightful subthemes, each shedding light on AI’s role in enhancing podiatric care. The following sections delve into these emergent themes.

### Theme 2.1: Distinction Between Supportive Tasks and Diagnosis

Nearly all participants agreed that AI should be used for support tasks such as triage. They highlighted that significant time is spent retrieving missing information not provided by patients and addressing issues with incorrect photo submissions that impede accurate assessment. They were of the opinion that these tasks could be efficiently managed by AI, thereby streamlining the process and improving overall efficiency. However, opinions varied on whether AI can diagnose foot conditions in patients with diabetes. Most participants were of the opinion that AI is incapable to do so, emphasizing the need for physical examinations by human experts to come up with an accurate diagnosis. They pointed out that photos fail to reveal crucial details, such as hidden wounds beneath calluses, which are not directly visible in pictures. Furthermore, participants noted the importance of physically assessing the feet for swelling and temperature variations (ie, warmth or coldness) that cannot be discerned from photos alone:

If it can highlight specific areas of concern, suggesting “pay attention here, this is alarming,” then I do place my trust in it.

Participant 3, female, age group 41-50 years

AI can’t make complete diagnoses; you need to hold the foot in your hands.

Participant 2, female, age group 41-50 years

I’m considering integrating a feature for predicting situations where an immediate consultation is crucial and distinguishing those from cases where it might not be as urgent.

Participant 1, male, age group 41-50 years

If it integrates well with practical needs, like photos can be easily uploaded directly into the file, making follow-up simpler. Yes, so it actually assists in your work.

Participant 6, female, age group 41-50 years

### Theme 2.2: AI as a Learning Tool

Participants acknowledged the potential of AI as a valuable learning tool, particularly for identifying unfamiliar diseases and suggesting innovative treatment strategies. They expressed optimism about AI’s ability to broaden their diagnostic and therapeutic horizons:

Maybe the AI will uncover a disease pattern I haven’t encountered before, offering me an opportunity to expand my knowledge.

Participant 1, male, age group 41-50 years

Hey, AI sees things differently, and that’s something I can learn from. I do see the added value in that.

Participant 5, female, age group 20-30 years

In this light, I believe that when an AI indicates, “this is the optimal treatment,” it becomes incredibly fascinating. It prompts a collective inquiry: “Why hadn’t this occurred to us before? Is this indeed the correct approach? Or is there already a hospital implementing this method?”

Participant 4, female, age group 31-40 years

### Theme 2.3: Reducing Workload Through AI

Participants highlighted the health care sector’s challenge of increasing workloads, worsened by a decreasing number of podiatrists and a growing number of patients with diabetes. There was widespread agreement on AI’s ability to alleviate this load. By automating processes such as triage, AI can significantly reduce the workload, freeing podiatrists to focus on the more crucial parts of patient care:

As the pressure continues to increase, I see AI as a great tool to indeed perform a kind of triage to ensure that the workload doesn’t become overwhelming.

Participant 3, female, age group 41-50 years

But yes, to be completely honest, looking towards the future, we definitely need to reduce the workload; otherwise, we’ll end up with even fewer people having to help more individuals.

Participant 4, female, age group 31-40 years

#### Theme 3: Factors Influencing Trust

### Overview

Finally, we delved into the pivotal elements that shape trust in AI-provided advice. We also probed when participants might be inclined to defer to AI’s judgment over their own. This investigation unveiled 3 subthemes, each shedding light on the interaction between humans and AI systems.

### Theme 3.1: Transparency and Explainability of AI

All participants agreed that clear explanations and transparency are essential, considering them as fundamental to trusting AI. They wished to comprehend the methodology AI uses to arrive at its recommendations. They proposed several methods to enhance transparency, such as providing concise explanations of the criteria AI uses for its evaluations, statistical performance data, or visual aids such as photos with overlaying layers or color coding:

I believe factors like the colour of the callus, the size of the area, its location, and history also play a role. Has there been a wound in that spot before? Yes, things like that.

Participant 8, male, age group 41-50 years

For example, I want to see a number of photos of the foot along with some explanation about them. I want to understand how the evaluation system is designed and the data it uses.

Participant 2, female, age group 51-60 years

It increases my confidence in knowing the sources from which AI retrieves its information or the ones I’ve provided to it.

Participant 1, male, age group 41-50 years

### Theme 3.2: Ensuring Reliability and Adequate Training

Participants were of the opinion that AI should be advanced enough from the beginning, emphasizing that a more extensive database of photos would increase their trust in the system. They would like AI to provide consistent advice, ideally on par with a fellow podiatrist. Training was highlighted as a crucial element for enhancing AI’s performance. In addition, understanding who the designers are and the data used for training is considered necessary. If participants know the designers are experts in their field, they feel more confident in the AI tool’s reliability:

Well, if you’ve only input three things into it, I won’t trust it. But if you’ve input 300,000 things, then at some point, you really know better.

Participant 2, male, age group 41-50 years

Well, I’m not quite sure how that works, but if that’s the case, then the people involved in setting it up need to have the right background knowledge, the right experience with the subject; they need to be specialists, so to speak.

Participant 2, female, age group 51-60 years

### Theme 3.3: The Value of Human Interaction

Participants emphasized that future AI applications must not compromise their interaction with patients, highlighting each patient’s uniqueness and background. They cautioned that AI may not always account for these personal nuances, underlining the importance of maintaining the human touch in health care:

I still believe that personal human contact will never disappear.

Participant 3, female, age group 41-50 years

All those kinds of things. Yes, they’re slightly different for everyone.

Participant 1, male, age group 41-50 years

But you still maintain human contact between the client and yourself, allowing them to easily ask additional questions, for instance, to you.

Participant 5, female, age group 61-70 years

#### Moving From Phase 1 to Phase 2

The findings from phase 1 revealed that AI can significantly improve various practical aspects of podiatry, particularly by alleviating workloads and serving as a learning tool. A clear preference was observed for using AI in supportive tasks rather than diagnostic roles. This reflects a cautious attitude of podiatrists toward AI-generated advice and raised the question: Will AI support be more readily accepted in supportive than in diagnostic tasks, and what factors influence this difference in acceptance?

Therefore, phase 2 of our study sought to explore the specific applications of AI in supportive versus diagnostic roles within podiatry in detail. Given the ongoing reluctance toward its role in diagnostics, this study aimed to understand the elements that can foster trust in AI for supportive tasks. By introducing a new group of podiatrists, phase 2 of our study aimed to gather fresh insights and assess whether the findings observed in phase 1 align with new professional perspectives.

#### Phase 2: Focus Group

##### Participants

Table 2 outlines an overview of the demographics of the focus group participants. The group consisted of 5 participants, 4 of whom were new podiatrists who engaged in the discussion. This group consisted of specialists in foot care for patients with diabetes, who primarily provide daily consultations to these patients.

**Table 2:** Participants’ characteristics from focus groups in phase 2 (n=5).

Characteristics			Participants, n (%)
**Sex**			
	Female	4 (80)	
	Male	1 (20)	
**Age group (y)**			
	41-50	2 (40)	
	51-60	2 (40)	
	61-70	1 (20)	
**Education level**			
	Bachelor’s	2 (40)	
	Master’s	3 (60)	
**Specialized in foot care for patients with diabetes**			
	Yes	5 (100)	
	No	0 (0)	
**Experience (y)**			
	20-30	4 (80)	
	31-40	1 (20)	
**Experience with the app**			
	Yes	2 (40)	
	No	3 (60)	

In phase 1 of our study, we opted for a flexible approach, allowing themes to emerge directly from the transcribed data. This exploratory phase enabled us to capture many insights directly from participants’ experiences and perspectives. Moving into phase 2, we shifted toward a more structured approach, using themes, which were relevant for phase 2 and predefined in phase 1. This transition allowed us to refine our analysis, focusing on the detailed examination of specific, predetermined themes to gain deeper insights and understanding of the topics at hand.

For a clear overview, we first present the key findings from the participants about various transparency levels for the 2 mock-ups in Table 3. Subsequently, in Table 4, we display the other themes carried forward from phase 1 for further analysis in phase 2, ensuring a comprehensive and structured exploration of the data.

**Table 3:** Thematic framework analysis for key findings and preferences of transparency levels in AI^a^ applications.

Theme: transparency and explainability of AI		Mock-up 1: AI support (triage and scheduling)	Mock-up 2: AI diagnosis (photo analysis)
**Low (variant A)**		Podiatrists perceive this variant as a “black box,” with unclear determination of urgency and relevance of the presented information	Feedback indicates general dissatisfaction and a lack of clarity on how AI reaches its conclusions
	First preference	—^b^	P4 and P5 (n=2)
	Second preference	—	—
	Third preference	P1-P5 (n=5)	P1-P3 (n=3)
**Medium (variant B)**		Podiatrists express concerns over the reliability of patient feedback as input for AI, pointing out a lack of crucial information such as wound location	While the text is deemed acceptable, this variant raises questions about the necessity of repeating information
	First preference	—	—
	Second preference	P1-P5 (n=5)	P1-P5 (n=5)
	Third preference	—	—
**High (variant C)**		Preference is given to detailed information, including specific medical details and clear patient identification	Podiatrists appreciate the clarity and added layer of understanding this variant provides but question the necessity of certain statistics, such as a reliability percentage
	First preference	P1-P5 (n=5)	P1-P3 (n=3)
	Second preference	—	—
	Third preference	—	P4 and P5 (n=2)

^a^AI: artificial intelligence.

^b^Not applicable.

**Table 4:** Thematic framework analysis for AI^a^ applications and predefined themes.

Themes	Participant 1 (female; age group 51-60 y)	Participant 2 (female; age group 41-50 y)	Participant 3 (male; age group 41-50 y)	Participant 4 (female; age group 51-60 y)	Participant 5 (female; age group 61-70 y)
Distinction between supportive tasks and diagnosis	Prefers AI for triage due to speed and accuracy. Skeptical about diagnostic capability without physical examination	—^b^	Values AI’s ability to prioritize photos based on urgency, enhancing patient care efficiency	Believes AI is incapable of handling complex diagnostic tasks, such as differentiating between types of wounds	Asserts that computers cannot replace human nuanced observation in diagnostics, emphasizing AI’s use in triage
Ensuring reliability and adequate training	Values the volume of feedback, similar to how reviews inform vacation choices	Trust is reinforced by a large volume of photos, indicating data reliability	Importance of data diversity, including training AI on poor-quality photos	Questions the repetitive mention of the photo database size suggest it is only necessary in training	Trust depends on knowing the AI’s advice is based on an extensive and diverse photo database
Long-term trust development	—	Trust in the algorithm grows over time with proven accuracy	Initial skepticism evolves into trust after multiple positive experiences	—	Anticipates a future shift in acceptance of AI’s advice, especially without a photo, within a decade
The value of human interaction	—	—	—	Asserts that physical tools and examination are indispensable for accurate diagnostics	Stresses the irreplaceable role of human involvement in making accurate diagnoses

^a^AI: artificial intelligence.

^b^Not applicable.

For the frameworks (Tables 3 and 4), instead of using quotes from focus group participants, we chose to provide summaries of the key findings, following the common practice in thematic framework analysis. By creating summaries, we were able to handle the complex discussion, which involved a record with 7 speakers, more easily.

##### Interpretations of Thematic Framework Analysis

On the basis of the thematic framework analysis of podiatrists’ preferences for AI integration in clinical settings, a clear pattern emerges regarding the desired application and form of transparency in AI systems for triage. Podiatrists prefer using AI in triage, emphasizing the importance of transparency that goes beyond just being open. They are looking for a kind of transparency that involves clinically relevant and detailed information—such as patient history, specific conditions such as the presence of vascular diseases, and exact wound locations—that significantly augment the decision-making process:

The added value for me is that the ranking is done by the AI, so photos are prioritised based on urgency.

Participant 3, male, age group 41-50 years

My preference is for triage; an AI can perform it much faster than a human. Well, I believe that AI is capable of making a risk assessment.

Participant 1, female, age group 41-50 years

Key abnormalities must be quickly recognised and addressed, achievable through triage.

Participant 5, female, age group 61-70 years

In the domain of triage, skepticism toward patient-reported data as the sole basis for AI-generated advice is evident (eg, medium transparency mock-up 1). The concern centers on the reliability of such data, highlighting a preference for AI systems that incorporate a comprehensive training dataset to inform their assessments. This reflects a broader apprehension regarding the accuracy of AI recommendations based on potentially incomplete or inaccurate patient self-assessments:

I’m not entirely convinced by the patients’ responses. If they say they have a wound on their feet, then yes, I might believe it, but if they don’t have a wound, I’m sceptical.

Participant 1, female, age group 41-50 years

Patients often do not have a clear understanding of complications.

Participant 3, male, age group 41-50 years

Podiatrists recognize the potential benefits but are cautious regarding the use of AI for diagnostic applications, particularly those involving the analysis of photographic evidence of foot conditions. This caution is based on the understanding that certain conditions cannot be fully diagnosed through images alone, emphasizing the need for AI systems to supplement rather than replace traditional diagnostic methods:

Computers can’t replace humans in diagnostics, especially not for tasks requiring nuanced observation, like identifying underlying issues beneath a callus that AI might miss.

Participant 5, female, age group 61-70 years

AI is incapable of making diagnoses. AI cannot handle complex conditions like distinguishing between warm and cold wounds.

Participant 4, female, age group 51-60 years

Even a human can’t make a diagnosis just from an image. The foot needs to be examined in person and potentially opened up for an accurate assessment.

Participant 1, female, age group 41-50 years

The desired level of transparency in diagnostic AI varies among podiatrists. While some value additional explanatory features, such as annotated images or textual diagnostics, others caution against the potential for such features to overly focus attention on a specific area, possibly leading to the overlooking of important symptoms in other areas. This division highlights the need for a careful balance in using AI; while it is beneficial to receive detailed, actionable insights, it is crucial to ensure that these insights do not overshadow the importance of using one’s own professional knowledge and experience in decision-making:

Works well for me; the layer aids in understanding the basis of the text.

Participant 3, male, age group 41-50 years

Also, here, the text is okay. I also like the layer, so now I know what the text is referring to.

Participant 1, female, age group 41-50 years

The explanatory text doesn’t interest me much; I’ll take it on faith what it’s based on.

Participant 4, female, age group 51-60 years

And that layer, I can see that for myself, too. Adding a layer can also be risky; I see even more things, and now AI dictates where I should look. A less experienced podiatrist might then focus solely on that.

Participant 5, female, age group 61-70 years

In addition to emphasizing transparency in integrating AI into clinical podiatry practice, our analysis has other predefined themes within the framework (Table 4). These themes enrich our understanding of how AI can be effectively applied in triage and diagnostics, emphasizing the need for clear, actionable insights and the importance of trust and data reliability as foundational elements. Reflecting on the preferences expressed by podiatrists, it becomes apparent that alongside transparency and explainability, considerations around data reliability, human interaction, trust development, and trust establishment align with sentiments from the first phase of our study.

**Figure 6:**
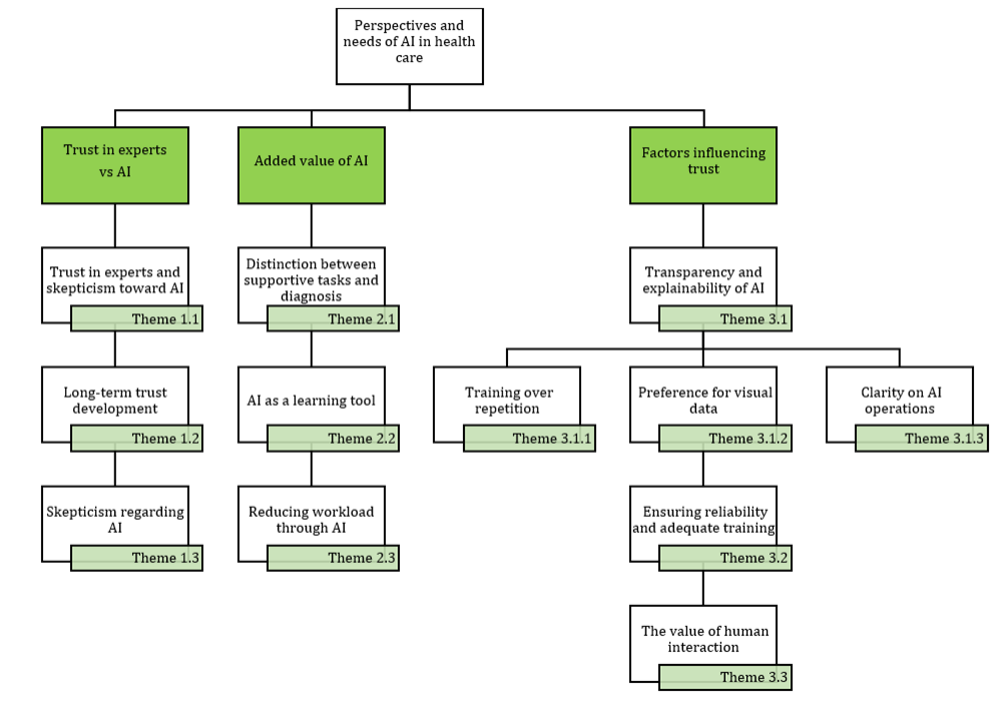
Generated themes and subthemes from phases 1 and 2: perspectives of experienced podiatrists on the FootCheck app and decision-making process. AI: artificial intelligence.

However, we have defined 3 additional themes that resonated strongly with the initial input for transparency and explainability. Figure 6 presents a comprehensive overview of all the themes, including the 3 subthemes (ie, 3.1.1: Training Versus Repetition; 3.1.2: Preference for Visual Data; and 3.1.3: Clarity on AI Operations) and their implications for integrating AI into podiatry. The following sections detail these additional subthemes further.

##### Subtheme 3.1.1: Training Versus Repetition

While participants in phase 1 primarily highlighted concerns among podiatrists regarding data reliability, phase 2 revealed a nuanced understanding of how and when this information should be introduced. This is particularly relevant during the initial training sessions where podiatrists are first taught how to use the AI system. Feedback on mock-up 2 indicated that emphasizing data reliability in every AI recommendation is not always seen as necessary. Some participants supported this viewpoint, while others preferred having this information included. In addition, there was consensus that explanations should offer insights beyond basic knowledge, aiming to augment understanding rather than merely repeat well-known information:

In the introduction (training), mention somewhere that the data is based on 5000 photos, but that doesn’t need to be in every piece of advice.

Participant 4, female, age group 51-60 years

I expect an AI to be backed by a vast photo database, so it’s unnecessary to reiterate that point for me.

Participant 5, female, age group 61-70 years

The text is helpful; it lets me know what the AI is based on.

Participant 3, male, age group 41-50 years

##### Subtheme 3.1.2: Preference for Visual Data

There is a significant inclination toward incorporating visual data alongside algorithmic risk assessments. Participants consider combining photos with textual advice crucial, enhancing their ability to make knowledgeable decisions. The need for visual confirmation to solidify trust in AI-generated advice is highlighted, with participants expressing reservations about relying entirely on text-based recommendations. Although color-coded elements are appreciated for their clarity, the facility to view photos directly via pop-ups is deemed essential for building confidence in the advice provided:

The colour coding in mock-up 1 is helpful, but I’d prefer to see the photos appear as pop-ups.

Participant 4, male, age group 41-50 years

Just text, without a photo, doesn’t inspire my trust.

Participant 3, female, age group 51-60 years

It seems like a small ask to quickly review the photo for a better trust in the advice.

Participant 4, female, age group 51-60 years

##### Subtheme 3.1.3: Clarity on AI Operations

Podiatrists highlight the necessity for transparency regarding the process behind AI-generated advice, which is essential for building trust and enabling users to understand when and how to rely on these recommendations. Opinions vary on the best way to present this information, with some finding objective data in photos and layered visuals helpful for understanding the advice’s basis, as seen in mock-up 2. Others question the interpretability of numerical data, such as “73%,” without seeing the patient in person:

Now, with a photo and layer, the information is more objective; you know what the text is about.

Participant 3, male, age group 41-50 years

I appreciate it, as then I know what it’s based on, mock-up 2.

Participant 1, female, age group 51-60 years

## Discussion

### AI in Supportive Roles Versus Diagnostic Tasks

Our study aimed to discern the multifaceted roles AI could assume in health care, specifically focusing on how AI can be effectively integrated into practice in a way that ensures health care professionals’ acceptance and trust in its advice. The findings from our studies suggest a preference for deploying AI in supportive roles. For instance, AI’s use in triaging processes is evident; by evaluating submitted photos for urgency, AI can advise health care providers in prioritizing patient consultations. This enables a more efficient allocation of resources, ensuring that patients requiring urgent care receive it promptly, while less-critical cases are appropriately prioritized. Such applications align with and enrich suggestions from the literature, including studies by Verma et al [10] and Longoni et al [5], suggesting the supportive use of AI to foster trust within health care settings. However, a contrasting perspective is presented in another experiment by Longoni et al [5], where preference leans toward human expertise over automated systems for triage tasks. This discrepancy from our findings may stem from differing viewpoints, with the study results of Longoni et al [5] being based on patient perspectives, whereas our study was performed among health care providers.

Another critical insight from our study highlights health care professionals’ hesitancy to embrace AI for diagnostic purposes, a sentiment that aligns with previous research findings [5,7,9,10,18]. While there is acknowledgment of AI’s proficiency in critical functions, such as making diagnoses, with results that match or even surpass human expertise, there remains skepticism among participants about AI’s current proficiency in performing these tasks accurately. The observed reluctance may stem from unfamiliarity with the full scope of AI’s capabilities, as evidenced by the fact that particularly participants without prior AI experience expressed reservations. Moreover, the conversations uncovered that identifying conditions such as diabetic foot complications poses distinct obstacles for AI. The participants pointed out that superficial evaluations, such as analyzing diabetic wounds via photographs, could be inadequate without the physical removal of calluses to reveal the actual problem, an action AI is incapable of without a physical examination. This underlines the importance of direct human interaction in specific diagnostic processes, highlighting limitations in the potential application of AI.

### Algorithm Aversion Among Podiatrists

The observed phenomenon of algorithm aversion among podiatrists, particularly their preference for human expertise over AI-generated recommendations, reflects a trend noted across various fields where AI competes with human judgment [26-29]. For example, Önkal et al [30] demonstrated a similar inclination among forecasters to favor expert opinions in forecasting decisions. A notable factor contributing to this aversion is the concern over mistakes made by AI systems [26], with our podiatrists voicing worries about the grave repercussions of such errors, including the risk of limb amputation. Although the significance of decisions ranges from health care to forecasting tasks in logistics, the underlying dread of AI-induced errors remains pervasive.

However, in our studies, this aversion was primarily directed at diagnostic tasks rather than supportive functions. Participants expressed confidence in the expertise and experience of their colleagues but also showed openness to considering AI-generated advice for tasks such as triage. This nuanced view suggests a complex relationship between podiatrists and AI, where trust may be contingent on the specific task at hand and the perceived reliability of AI in enhancing rather than replacing human judgment.

### Transparency and Explainability

The discussions surrounding transparency and explainability in our studies mirrored the diversity of perspectives found in the broader literature on these subjects [12,14,19]. The need for explanation and transparency depends significantly on podiatrists’ individual preferences. Our study provides 3 key insights regarding the implementation of explainability and transparency in AI systems.

First, precise and valuable explanations that avoid redundancy are essential for enhancing user understanding and trust in AI. Second, visual aids alongside textual advice can boost confidence in AI recommendations more than text alone, by making the motivation behind complex decisions more accessible. Finally, it is important to remind users to balance AI advice with their professional judgment, highlighting the crucial role of human oversight in AI decision-making.

However, these discussions were particularly animated regarding the scenario involving AI-generated diagnoses, where participants displayed heightened criticality toward the information received from AI. This suggests that individuals are particularly cautious about relying on AI for diagnostic tasks, scrutinizing the transparency and explainability of such systems with greater intensity. Interestingly, these issues were less extensively debated in contexts involving AI for supportive tasks. This observation highlights an area for future research.

### Development of Trust Over Time

Most participants indicated a higher level of trust in their colleagues than in AI systems, suggesting that trust in AI might evolve positively with increased exposure and evidence of AI’s efficacy. However, this remains speculative, as our participants have not extensively experimented with AI in their professional practice. The notion that repeated interactions with AI could enhance trust aligns with existing literature [31,32]. This area, highlighting the dynamic interplay between growing familiarity with AI and the shifting landscape of trust and reliance, remains underexplored in our studies and represents a fruitful avenue for further research.

### Strengths and Limitations

Our research is strengthened by several methodological approaches that enhance the credibility of our findings. The employment of 2 independent researchers for parallel data coding in both studies significantly contributed to the reliability of our results, as evidenced by the substantial agreement in their coding outcomes [33].

The relatively small sample sizes of 9 podiatrists in the first phase and 5 in the second phase may seem to be a limitation for generating general conclusions. However, qualitative research often reaches a point of data saturation, where small samples are already sufficient to uncover dependable themes. In our study, we observed that no new codes or themes emerged after 7 interviews in the first phase, indicating that data saturation had been reached. This aligns with the findings of Guest et al [34], who noted that most codes are identified within the first 6 interviews. Guest et al [35] also supported this by providing a systematic method for assessing saturation, further validating our sample size.

The significant expertise of the podiatrists involved, that is, all specialists in the area considered, adds depth to our insights and strengthens the credibility of our findings. In addition, the second phase, with mostly new participants, confirmed the results of phase 1. By focusing on how AI might be more accepted in supportive tasks compared with diagnostic tasks, we were able to further validate the earlier findings and ensure that no new themes emerged, indicating that saturation had been reached.

### Conclusions

In conclusion, the findings reveal podiatrists’ complex attitudes toward AI in health care, demonstrating openness to AI assistance for supportive tasks while being cautionary regarding its application for diagnostics. This underscores the necessity for developers and policy makers to prioritize trust-building strategies, starting with opportunities that allow podiatrists to experience firsthand how AI can support their role. Such initial experiences could clarify AI’s benefits and capabilities to users, reduce perceived threats, and facilitate a smoother transition toward acceptance. With a foundation of trust established through the application of AI for supportive tasks, podiatrists may become more receptive to exploring AI’s potential for diagnostic tasks. Gradually expanding AI’s role from foundational tasks, including decision support, to areas requiring deeper trust, such as diagnostics, could enhance acceptance and integration of AI technologies in podiatric practice.

## Appendix

Multimedia Appendix 1 Interview guide used in phase 1.

Multimedia Appendix 2 Mock-ups used in phase 2.

Multimedia Appendix 3 Interview guide used in phase 2.

## Abbreviations

AI: artificial intelligence
XAI: explainable artificial intelligence

## Data Availability

The corresponding author will make the datasets supporting these studies available upon reasonable request.
