# Supplementary material for: Prioritizing Trust in Podiatrists’ Preference for AI in Supportive Roles Over Diagnostic Roles in Health Care: Qualitative Interview and Focus Group Study
Source: JMIR Hum Factors. 2025 Feb 21;12:e59010. doi: 10.2196/59010 (PMC11890136; doi:10.2196/59010)
Supplement: Multimedia Appendix 1 [file humanfactors_v12i1e59010_app1.docx]

| **Interview guide phase 1. Central questions and topics.** | |
| --- | --- |
|  | |
| **1. Opening questions**  - General perception of AI | 1. What do you already know about AI (advice)? For example, the suggestions you get from Netflix for movies and series or within healthcare, such as Skin Vision. 2. Have you experienced working with AI? If yes, how did you experience this? |
| **2. Trust in humans and AI advice**  - Personal sensitivity to trust in AI  - Human vs. AI advice   - Algorithm Aversion/Appreciation | 1. What are your general views and feelings about trusting AI? 2. How would you describe your trust in AI? 3. How do you feel about advice from a human (fellow podiatrist) compared to an A.I.? Why is that? |
| **3. AI's added value to daily tasks**   - Task size - High/low risk - Task complexity | 1. In which situations would you be more inclined to trust advice from a fellow podiatrist, and in which situations would you be more inclined to trust advice from an AI? 2. Can you briefly describe your activities as a podiatrist regarding diabetes foot care and the FootCheck app? 3. Can you provide concrete examples of how AI could add value to your (daily) work? |
| **4. Factors affecting trust in AI**  - Understandability  - Communication/education  - Explainability  - Transparency | 1. Under what specific circumstances or conditions would you be willing to set aside your professional judgment or advice instead of opting for an AI-generated recommendation? 2. To what extent would you find understanding an AI's workings essential for your trust in it? 3. What information would you like to receive to understand an AI and its advice better? 4. How important do you find transparency in the use of AI? Can you explain why? |
| **Closing** | Do you want to add anything to this conversation? Are there things necessary to mention that have not been covered in the conversation?  Thank you for your time and participation in the interview.  I hope you feel that your story has been listened to. |
