# Supplementary material for: Prioritizing Trust in Podiatrists’ Preference for AI in Supportive Roles Over Diagnostic Roles in Health Care: Qualitative Interview and Focus Group Study
Source: JMIR Hum Factors. 2025 Feb 21;12:e59010. doi: 10.2196/59010 (PMC11890136; doi:10.2196/59010)
Supplement: Multimedia Appendix 2 [file humanfactors_v12i1e59010_app2.docx]

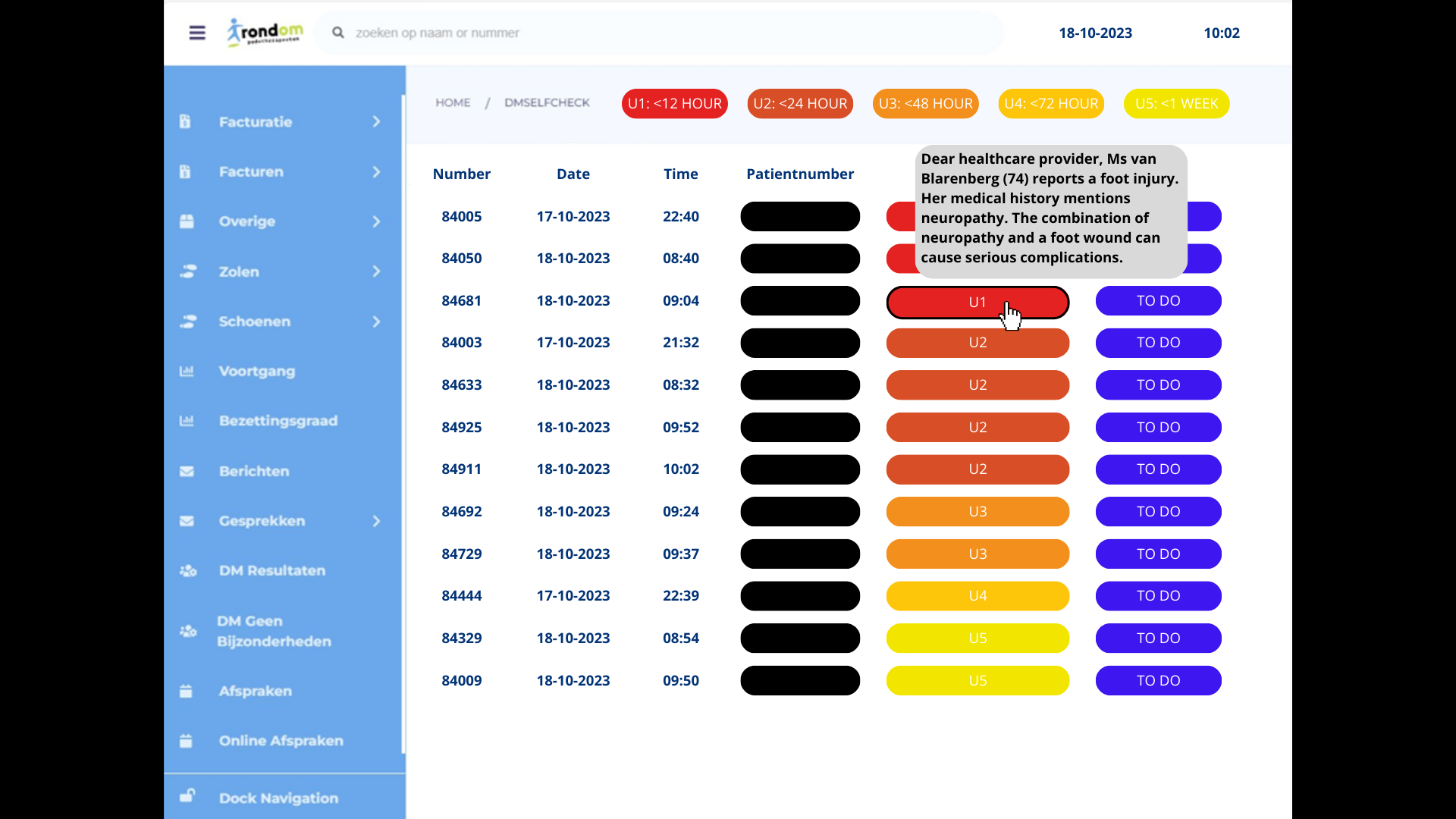

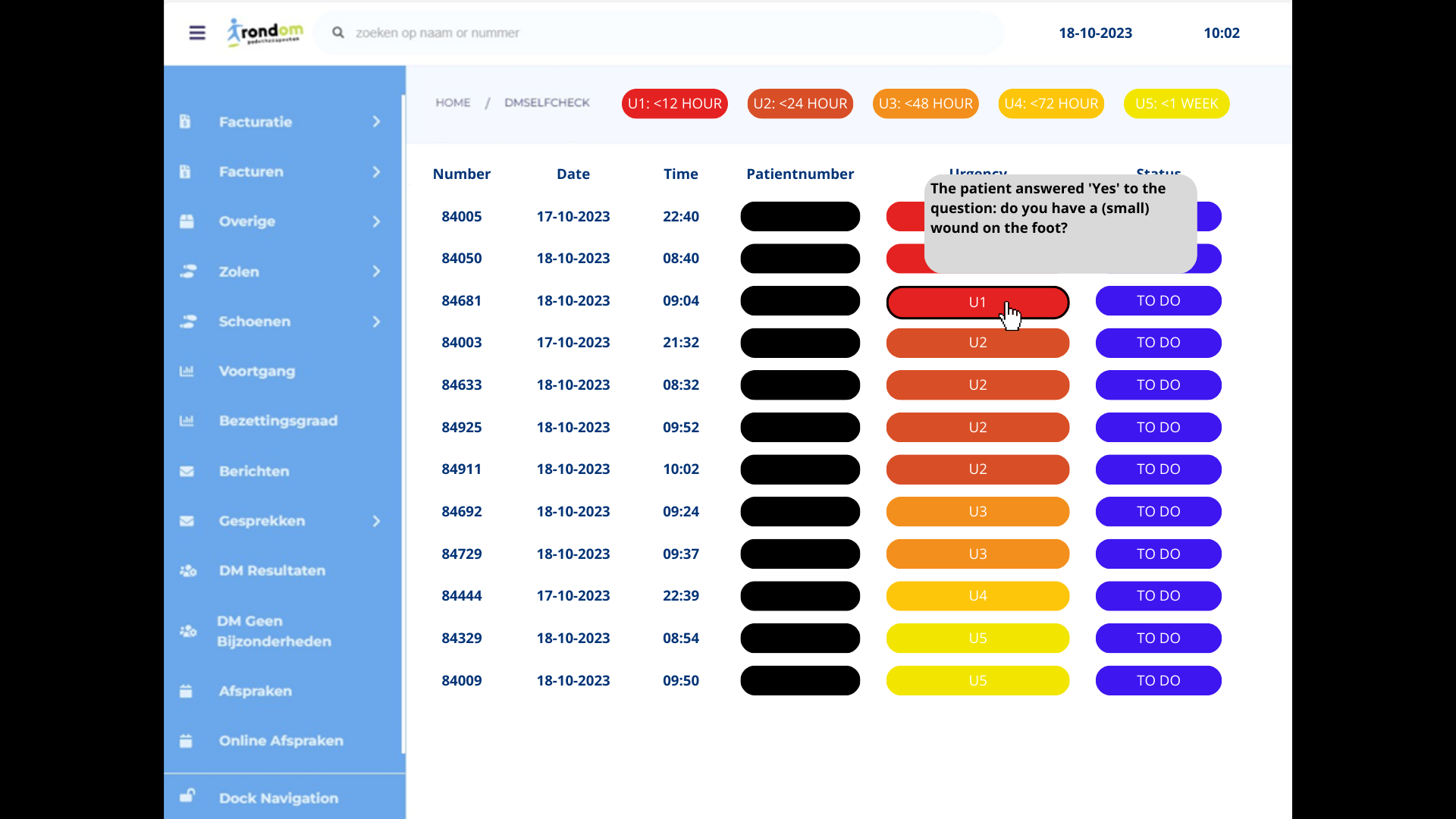

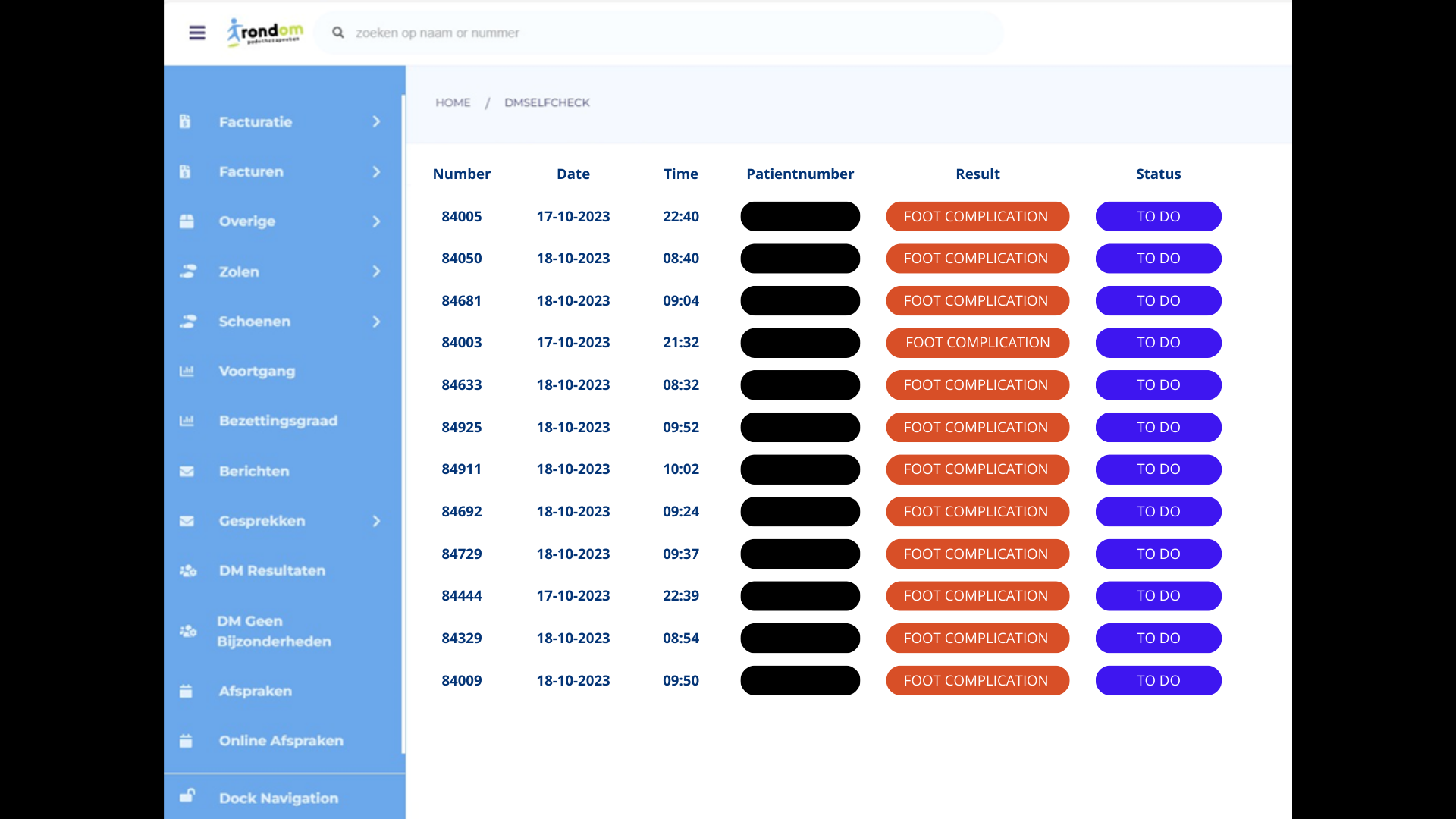


Hihg transparency (Variant C)

Medium transparency (Variant B)

Low transparency (Variant A)


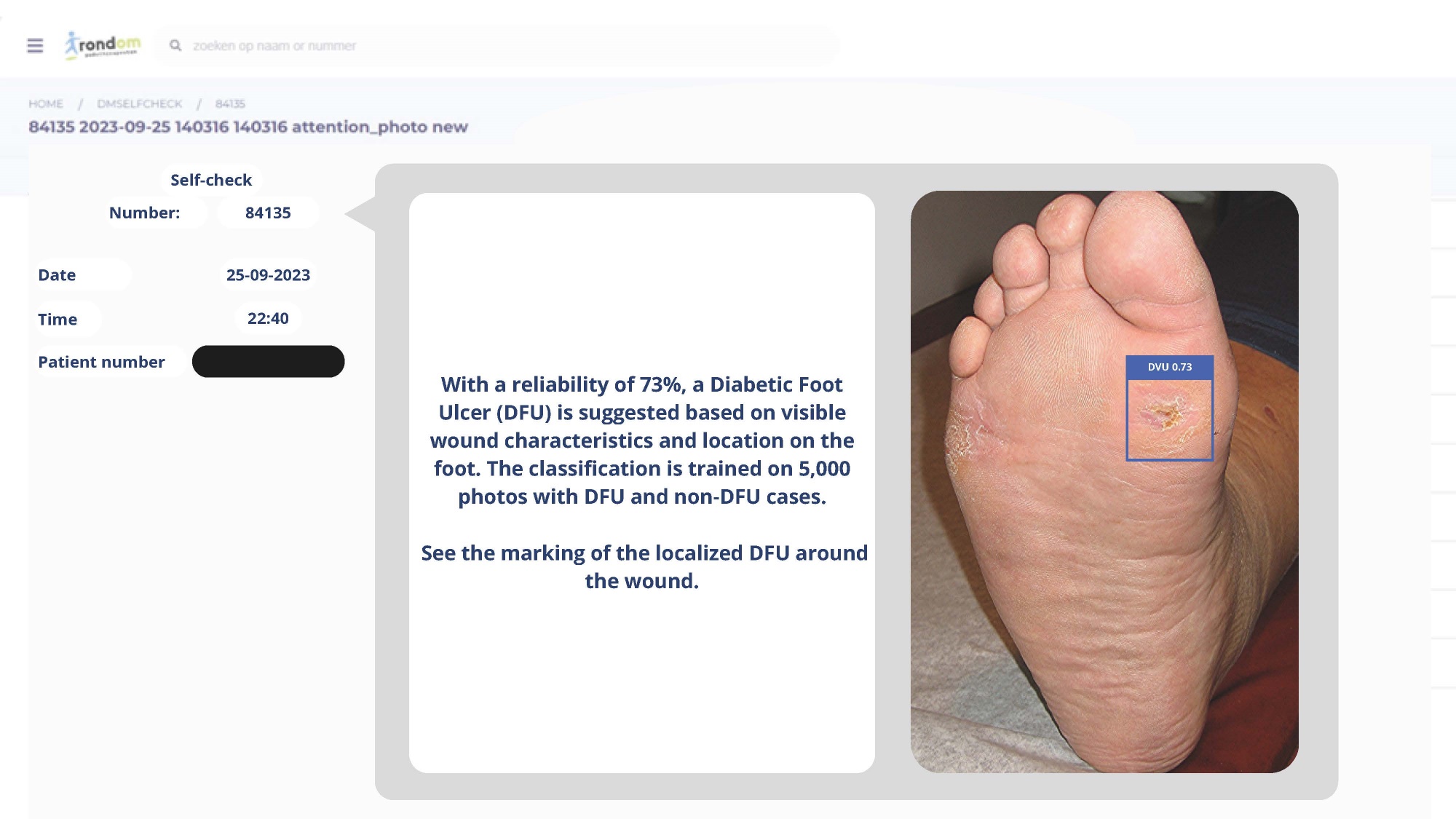

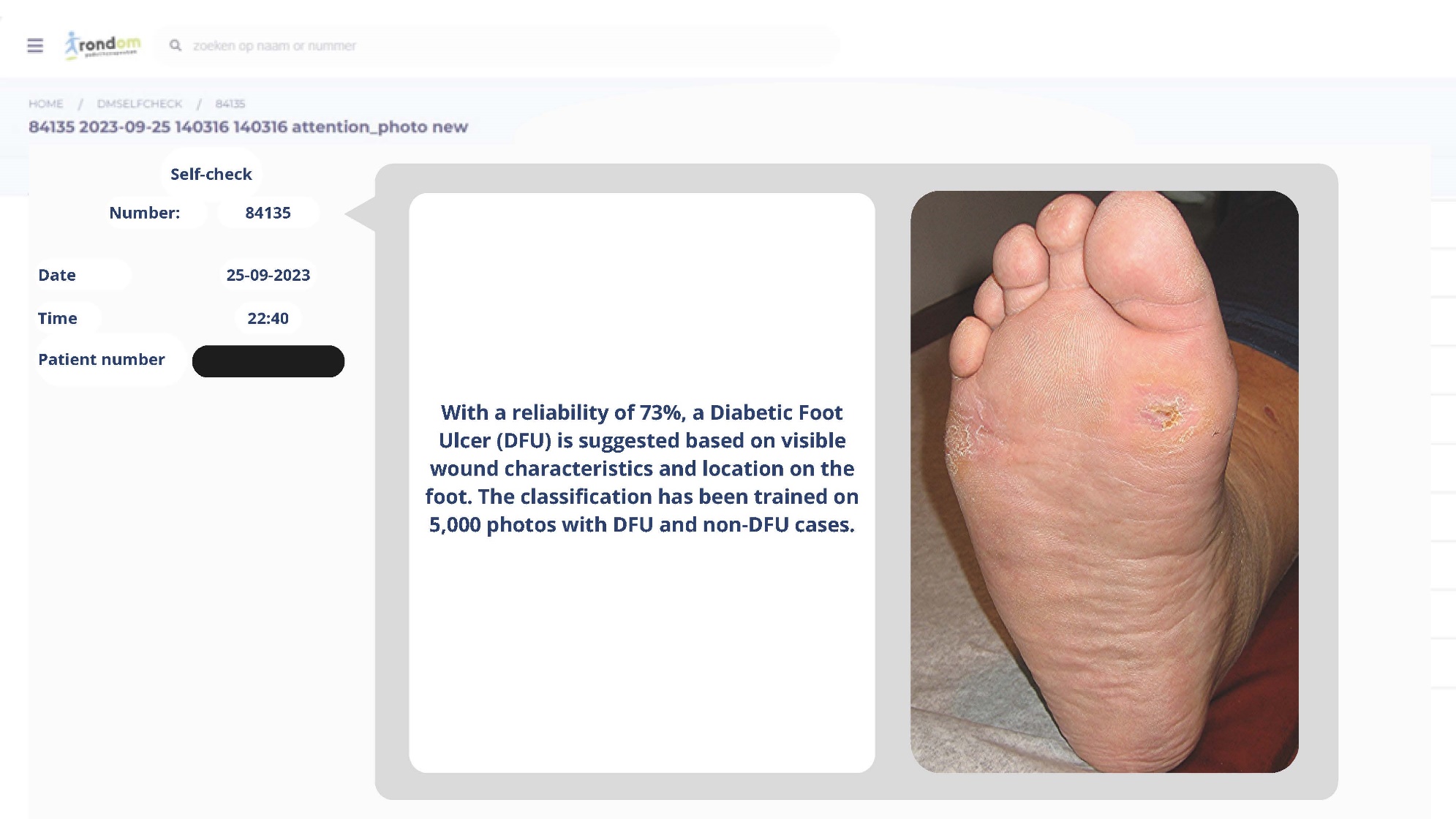

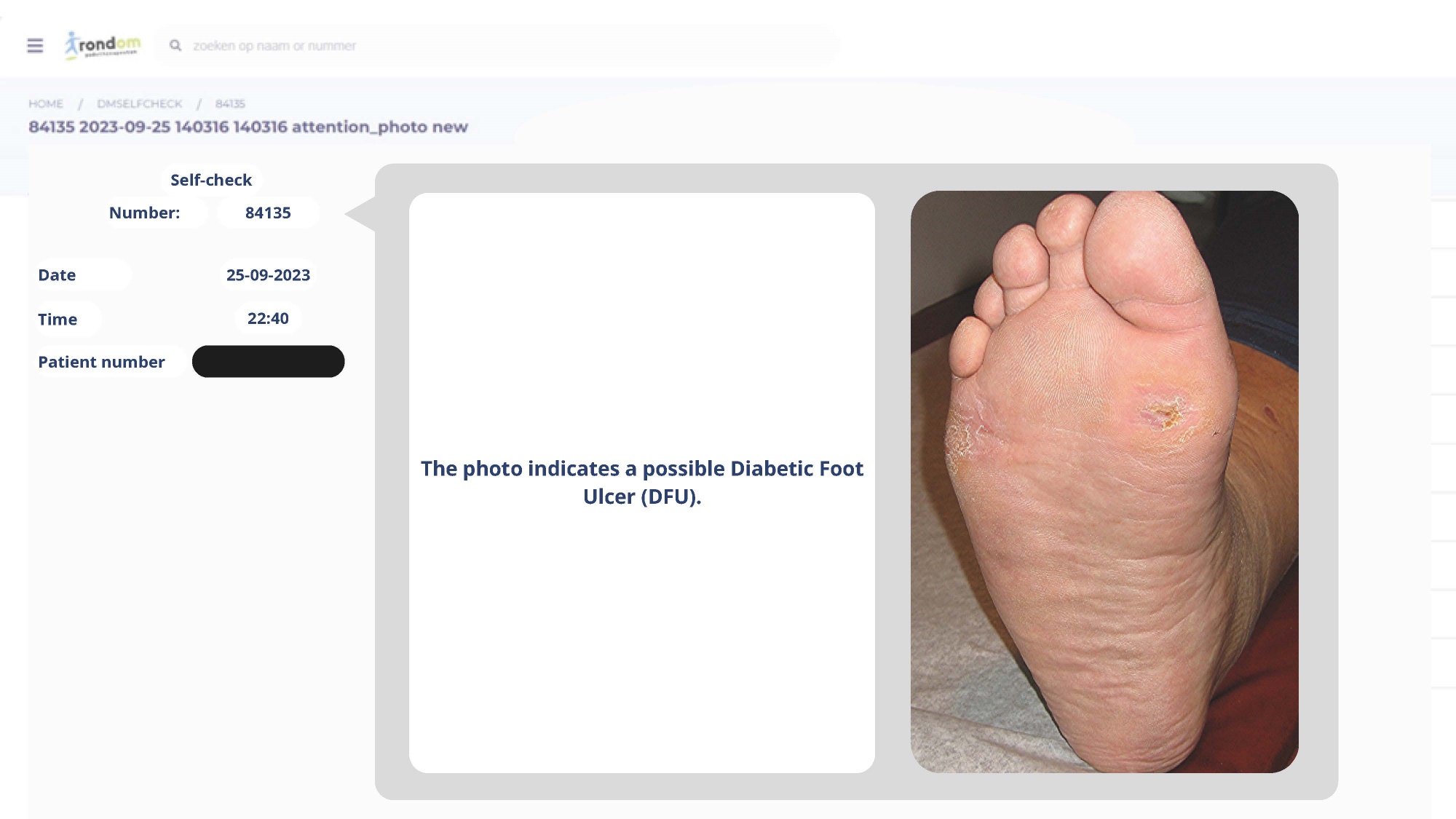


Hihg transparency (Variant C)

Medium transparency (Variant B)

Low transparency (Variant A)
