# Supplementary material for: Prioritizing Trust in Podiatrists’ Preference for AI in Supportive Roles Over Diagnostic Roles in Health Care: Qualitative Interview and Focus Group Study
Source: JMIR Hum Factors. 2025 Feb 21;12:e59010. doi: 10.2196/59010 (PMC11890136; doi:10.2196/59010)
Supplement: Multimedia Appendix 3 [file humanfactors_v12i1e59010_app3.docx]

| **Interview guide phase 2. Central questions and topics.** | |
| --- | --- |
|  | |
| **1. Introduction** | 1. Thank you for your willingness. We will soon present two mock-ups to you and would like to discuss them. First, please take some time to think quietly and write down your findings. Afterwards, we will engage in a discussion together. |
| **2. Trust in AI's role**  - Supportive (triage)  - Diagnostics | 1. Could you share your thoughts on which of the two scenarios (supportive vs. diagnostic) you would be more inclined to accept AI involvement and trust its advice, and why? |
| **3. Transparency preferences**   - Low, medium and high - Layers - Colours - Visuals (photos) - Short text | 1. Which transparency preference do you find most desirable, and why? (ask again after reviewing mock-up 2.) |
| **4. AI's effect on decision-making**  - Data reliability  - Training (data)  - Human-machine interaction | 1. Are there any other factors that, in your opinion, influence trust and acceptance? |
| **Closing** | Do you want to add anything to this conversation? Are there things necessary to mention that have not been covered in the conversation?  Thank you for your time and participation in this focus group discussion.  I hope you feel that your story has been listened to. |
